# The Deep Mining Era: Genomic, Metabolomic, and Integrative Approaches to Microbial Natural Products from 2018 to 2024

**DOI:** 10.3390/md23070261

**Published:** 2025-06-23

**Authors:** Zhaochao Wang, Juanjuan Yu, Chenjie Wang, Yi Hua, Hong Wang, Jianwei Chen

**Affiliations:** 1 College of Pharmaceutical Science & Collaborative Innovation Center of Yangtze River Delta Region Green Pharmaceuticals, Zhejiang University of Technology, Hangzhou 310014, China; 211122070145@zjut.edu.cn (Z.W.); 221123070219@zjut.edu.cn (J.Y.); 211123070063@zjut.edu.cn (C.W.); huayi@zjut.edu.cn (Y.H.); 2 School of Pharmaceutical Sciences, Guangzhou University of Chinese Medicine, Guangzhou 510006, China

**Keywords:** genomics, metabolomics, secondary metabolites

## Abstract

Over the past decade, microbial natural products research has witnessed a transformative “deep-mining era” driven by key technological advances such as high-throughput sequencing (e.g., PacBio HiFi), ultra-sensitive HRMS (resolution ≥ 100,000), and multi-omics synergy. These innovations have shifted discovery from serendipitous isolation to data-driven, targeted mining. These innovations have transitioned discovery from serendipitous isolation to data-driven targeted mining. Genome mining pipelines (e.g., antiSMASH 7.0 and DeepBGC) can now systematically discover hidden biosynthetic gene clusters (BGCs), especially in under-explored taxa. Metabolomics has achieved unprecedented accuracy, enabling researchers to target novel compounds in complex extracts. Integrated strategies—combining genomic prediction, metabolomics analysis, and experimental validation—constitute new paradigms of current “deep mining”. This review provides a systematic overview of 185 novel microbial natural products discovered between 2018 and 2024, and dissects how these technological leaps have reshaped the discovery paradigm from traditional isolation to data-driven mining.

## 1. Introduction

Natural products (NPs) have long been a key source of therapeutic lead compounds, with microbial natural products alone accounting for approximately 35% of FDA-approved small molecule drugs since 1981 [1,2]. Representative drugs in this category include antibiotics (penicillin), immunomodulators (cyclosporine), metabolic modulators (lovastatin), and others, underscoring their enduring relevance in drug discovery [3,4,5]. However, the decline of traditional “one-strain, one-compound” screening at the beginning of the 21st century has led to a pronounced bottleneck in microbial natural product discovery, prompting calls for technological innovations to explore untapped chemical diversity [6,7].

Over the past decade, synergistic advances in genomics, metabolomics, and numerous integrative strategies have driven a paradigm shift toward data-driven deep mining [8,9,10,11]. PacBio HiFi (long read accuracy > 99.9%) and Nanopore MinION (portable real-time analysis) have enabled microbial comprehensive genome analysis, revealing that only approximately 10% of the biosynthetic gene clusters (BGCs) in *Streptomyces* are expressed under standard culture conditions [12,13,14]. At the same time, metabolic detection technologies represented by ultrasensitive mass spectrometry and structural analysis technologies represented by cryogenic nuclear magnetic resonance (600 MHz + magnets), crystal sponge technology, nuclear magnetic resonance (NMR) calculations, and ECD spectroscopy are now capable of detecting trace metabolites and resolving complex stereochemistry, respectively, addressing historical limitations in structural analysis [15,16,17,18,19].

**Figure 1:**
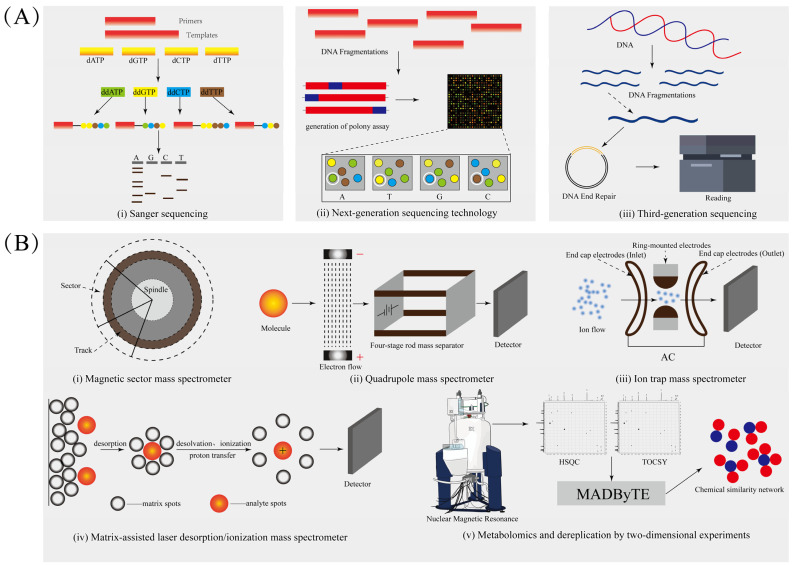
(**A**) Fundamentals of gene sequencing from the first to the third generation. (i) Sanger sequencing operates on the principle of selective chain termination during DNA replication; (ii) Next-generation sequencing is based on large-scale parallel sequencing and sequencing-by-synthesis; (iii) Third-generation sequencing technologies perform direct single-molecule sequencing of DNA/RNA, featuring long read lengths. (**B**) The basic principle of action of a variety of mass spectrometers. (i) Magnetic sector mass spectrometer employs magnetic field deflection to differentiate ion masses; (ii) Quadrupole mass spectrometry employs oscillating and static electric fields to selectively transmit ions based on their *m*/*z* ratios; (iii) Ion trap technology utilizes oscillating electric fields to spatially confine charged particles, followed by mass-dependent ejection through controlled destabilization of ion trajectories; (iv) Matrix-assisted laser desorption/ionization represents a soft ionization technique in which pulsed laser irradiation of analyte-embedded matrix crystals induces desorption and ionization of intact macromolecules for mass spectrometric detection; (v) The MADByTE platform employs TOCSY and HSQC spectral data to characterize composite spin systems and construct chemical similarity networks across sample cohorts.

Genome mining enabled the transition from phenotype-driven segregation to computerized prediction, revolutionizing NPs discovery. From the 1970s, with φX174-based Sanger sequencing, to today’s evolution into modern bioinformatics techniques utilizing machine learning and comparative genomics, rapid advances in the field of computers have begun to drive the reconfiguration of natural product chemistry technology [20,21,22,23,24,25]. The basic principle of sequencing is shown in Figure 1. Platforms like anti-smash 7.0 (2023) integrating hidden Markov models (HMMs) and artificial intelligence have expanded the number of annotatable BGC types to more than 40, while DeepBGC (2024) uses bi-directional long and short-term memory networks (BiLSTM) and Random Forests to identify orphan clusters in under-explored phyla (e.g., verrucose microbes) [26,27,28,29,30,31,32]. Key to this progress is multi-tool synergies, such as the combination of PRISM 2.0 for ribosomal peptide prediction with ClusterFinder for the analysis of polyketide-non-ribosomal peptide hybrids, which increased structural diversity coverage by 40% compared to single-tool analyses [33]. It is foreseen that next-generation sequencing (NGS) will further accelerate the development of this field [34,35]. If the cost of genome sequencing can be reduced by 99% within 15 years, the realization of large-scale projects like The Global Ocean Microbiome (GOMC) will no longer be limited by the high consumption of funds. Such large-scale metagenomic analyses could very quickly accelerate the discovery of cryptic BGCs encoding novel chemical skeletons, such as cyclopropane-ether hetero-polyketides from soil-derived *Microcystis aeruginosa*, validated via CRISPRi-mediated pathway activation [36].

As opposed to genomics, metabolomics has become another key enabler driven by separation and spectroscopic analysis techniques. The widespread adoption of high-resolution mass spectrometry platforms, including orbital trap, time-of-flight (TOF), and Fourier-transform ion cyclotron resonance (FT-ICR) systems, has dramatically improved detection sensitivity and mass measurement accuracy in metabolomics studies [37,38,39,40]. The working principle of various mass spectrometers is shown in Figure 1. Advances have also been made in NMR technology—2D NMR (COSY, HSQC) combined with cryogenic probes has increased signal sensitivity by 30%, enabling the stereochemical identification of marine diterpenes such as eunicellane, a feat unattainable with NMR technology a decade ago [41,42,43,44]. The launch of GNPS (Global Natural Products Social Molecular Networking) in 2016 marked a turning point, enabling community-wide sharing of MS/MS data to build metabolite association networks [45]. Combining feature-based molecular networking (FBMN) based on the GNPS platform with artificial intelligence tools (SIRIUS for molecular formula prediction and DeepMass for structure elucidation), researchers can now annotate unknown constituents in *Streptomyces* extracts with up to 65% higher accuracy than database-dependent methods, even for non-model strains [46,47,48].

The advantage of multi-omics integration in in-depth mining lies in the following: genomics reveals the strain’s potential for active product production, metabolomics captures specific secondary metabolites, and the combination of the two realizes a comprehensive analysis of biological systems from genes to phenotypes. Early integration approaches focused on correlation analysis to determine statistical associations between gene expression profiles and metabolite levels [49,50]. Nowadays, after genomics analysis, it is preferred to select high-value BGCs for subsequent product studies, such as the discovery of Mandimycin, through heterologous expression or One Strain Many Compounds (OSMAC) strategies [51,52,53,54]. This integration strategy addresses the “genome-metabolome gap”—where only 25% of predicted BGCs have known products—and strengthens the link between genomic BGCs data and real chemical objects. In this paper, we categorize the integration strategies into two types: those based on the combination of NMR and genomic analysis, and those based on the combination of liquid chromatograph mass spectrometer (LC-MS) and genomic analysis, show the scope of their applications, and highlight recent advances in combinatorial strategies for mining new NPs.

## 2. Genome Mining for NPs

### 2.1. Genome Mining of RiPPs

Genomic analysis of ribosomally synthesized and post-translationally modified peptides (RiPPs) places significant emphasis on post-translational modifications (PTMs), with RiPP family classification typically determined by their signature PTM enzymes [55]. A notable development in this field is the emergence of aromatic amino acid-containing RiPPs as a novel subclass, where the formation of specific C-C or C-N bonds during PTMs has been associated with P450 enzymes [56,57,58,59]. However, conventional genomics tools lack the capability to establish precise correlations between P450 enzymes and their corresponding RiPP products, necessitating the development of more innovative approaches to systematically identify P450 enzyme-modified RiPPs.

**Figure 2:**
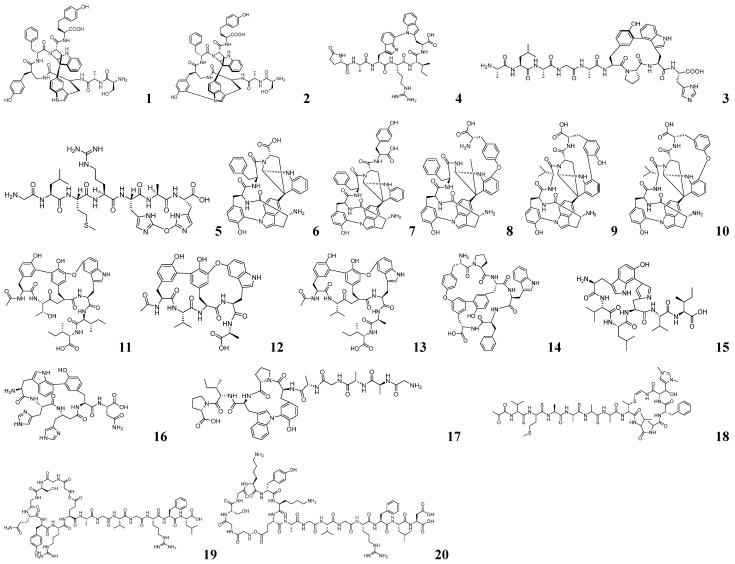
The chemical structures of RiPPs by genome mining (**1**–**20**).

The discovery of P450-associated RiPPs was achieved through an integrated workflow combining three advanced bioinformatics approaches: multilayer sequence similarity network (MSSN) for analyzing functional correlations among biomolecules, short peptide and enzyme co-localization (SPECO) for genome mining of RiPP BGCs, and AlphaFold-Multimer for predicting protein complex structures [60]. This comprehensive strategy began with SPECO analysis of 20,399 actinomycete genomes to identify potential PTM-involved sequences, followed by AlphaFold-Multimer structural predictions that revealed a conserved binding mode where precursor peptides embed their C-termini within P450 pockets while extending core peptides toward the heme center—a molecular signature enabling discrimination between authentic RiPP precursors and non-RiPP sequences. [61]. The workflow further incorporated MSSN construction of precursor peptide–P450 pairs, validated using established tryptorubin A and cittilin A families as reference datasets, which successfully identified three known and three novel RiPP families. Heterologous expression of four selected BGCs (*kst*, *mci*, *scn*, and *sgr*) in *E. coli* yielded five structurally diverse macrocyclic peptides, namely kitasatide 1019 (**1**), kitasatide 1017 (**2**), micitide 982 (**3**), strecintide 839 (**4**) and gristide 834 (**5**). The structures of these five compounds as shown in Figure 2. Biochemical characterization revealing that KstB, ScnB, and MciB are substrate-mixed P450 enzymes, which can achieve catalytic cyclization of unnatural precursor peptides and targeted substitution of specific amino acid residues, exhibiting great potential in the field of enzyme engineering. This method utilizes RiPP biosynthetic principles combined with multidimensional sequence-structure analyses to effectively distinguish between RiPPs-associated and non-associated P450s, thereby enhancing the precision and throughput of novel P450 discovery while expanding the target landscape for RiPP research.

**Figure 3:**
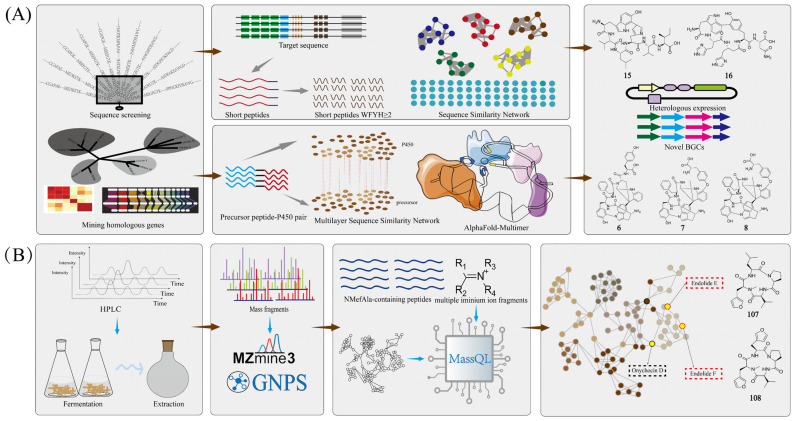
(**A**) Examples of genome mining for new NPs. (**B**) Examples of metabolomic mining for new NPs.

The identification of P450-modified RiPPs was facilitated through a streamlined bioinformatics pipeline integrating multiple computational tools. Initial sequence analysis employed BlastP for homologous protein identification and the Enzyme Function Initiative-Enzyme Similarity Tool (EFI-EST) for generating sequence similarity networks (SSNs), which enabled BLAST-based similarity analysis and network visualization [62]. RiPPer is a standardized prediction program based on ribosome binding sites (RBSs), specifically designed for predicting, analyzing, and studying RiPPs [63,64]. The rational use of known P450 sequences can also establish the correlation between P450 enzymes and RiPPs. Using three characterized P450 enzymes (BytO, CitB, and TrpB) as query sequences, Hui-Ming Ge and his team performed comprehensive database mining through BlastP searches against NCBI followed by EFI-EST analysis, yielding 13,896 non-redundant P450 sequences after length filtering [65]. The approximate mining process is shown in Figure 3A. RiPPer predicted potential precursor peptide sequences adjacent to P450 enzymes, focusing on those with multiple conserved aromatic amino acids. Combined with SSN analysis, 1057 P450-modified RiPPs gene clusters were successfully identified and classified into 11 categories. Heterologous expression of five novel BGCs, namely *tsu*, *oli*, *san*, *cre*, and *syr*, in *S. albus* J1074 yielded nine new compounds: tsukirubins A–C (**6**–**8**), olivorubins A–B (**9**–**10**), shandoamide (**11**), syrinamides A–B (**12**–**13**), and citreamide (**14**), which are shown in Figure 2. This study uncovered 11 new P450-modified RiPPs from four classes via genome mining, broadening structural diversity [65]. A simple and efficient RBS-based workflow was established for identifying P450-modified RiPPs gene clusters. Unlike complex prediction tools, it utilizes RBS as the identification label and integrates SSN analysis, significantly reducing computational effort while improving accuracy and efficiency.

Position-Specific Iterated Basic Local Alignment Search Tool (PSI-BLAST) detects tele-evolutionary relationships between proteins, identifies conserved structural domains or motifs in protein sequences, and optimizes the search results by iteratively constructing a position-specific scoring matrix [66]. Rapid ORF Description and Evaluation Online (RODEO) is a bioinformatics tool designed for the rapid identification and evaluation of open reading frames (ORFs) [67]. It is particularly well-suited for predicting potentially functional ORFs from metagenomic or transcriptomic data. Seokhee Kim and his team utilized these two tools to establish the connection between P450 and RiPPs [68]. The approximate mining process is shown in Figure 3A. Using TrpB (associated with aromatic-containing peptides) and BytO (linked to coaromatic peptides) as query sequences, researchers employed PSI-BLAST to identify evolutionarily related P450 homologs, while RODEO facilitated efficient prediction of adjacent ORFs encoding potential precursor peptides with characteristic aromatic features. This dual-tool strategy enabled the identification of 19 novel BGCs through PSI-BLAST analysis. Three of the precursor peptides predicted by these BGCs have rare aromatic amino acid residues and remain well-translated when co-expressed with the corresponding P450 enzymes. Following heterologous expression of the BGCs corresponding to the three selected precursor peptides in *E. coli*, three new modified peptides, namely roseovertin (**15**), rubrin (**16**) and lapparbin (**17**) were obtained, which are shown in Figure 2. These findings led to the establishment of “cyptides” as a new RiPP superfamily, defined by their characteristic P450-mediated multi-linkage aryl cross-bridges(Trp C7′-to-His N-τ, Tyr C-6-to-Trp N-1′, and Trp C-7′-to-Tyr C-6), which substantially expands the structural diversity of post-translationally modified peptides. This method demonstrates how strategic integration of PSI-BLAST’s sensitive homology detection with RODEO’s efficient ORF prediction can effectively make a connection between P450 enzymes and their cognate RiPP products, providing a robust framework for future discovery of complex peptide modifications.

The integration of diverse bioinformatics tools facilitated the establishment of precise correlations between P450 enzymes and RiPPs, enabling the systematic mining of such RiPPs. Screening for the genes with high similarity or homologs as target genes is an efficient approach for identifying other members of a compound family. The genes identified through screening often fail to express the target product under laboratory culture conditions and typically require heterologous expression or genetic modification [51,69,70].

The *λ*-Red system, derived from the *λ*-Red phage, overcomes the limitation of restriction endonuclease cleavage sites through homologous recombination technology [71,72]. It is widely used for various genetic modifications and is one of the most common tools in gene editing. Kazuo Shin-ya and his team employed BLAST analysis to identify a precursor peptide gene containing the VMAAAATVAFHC motif in *Streptomyces* sp. MSB090213SC12, showing high similarity to the known thioredoxin sequence (VMAAAASIALHC) within a genome database comprising over 100 actinomycetes [73]. The target neothiocyanin biosynthetic gene cluster (*ntv*) was isolated through the construction of a Sau3AI-digested BAC library and subsequent PCR screening, yielding the clone pKU503J143P1-J10. Minimized gene clusters were obtained following modification of the cloned genes using the *λ*-Red system. These optimized clusters were heterologously expressed in *S. avermitilis* SUKA22, leading to the production of the novel thioredoxin compound neothioviridamide (**18**), which is shown in Figure 2. The approach demonstrates how combining computational prediction with advanced genetic manipulation can unlock the biosynthetic potential of silent gene clusters that remain refractory to conventional fermentation-based discovery methods.

Genetic annotation of the fungal-bacterial endosymbiotic system *Mycetohabitans rhizoxinica*, conducted by Christian Hertweck and his team, revealed its potential to produce burhizin and mycetohabin-like lasso peptides [73]. Initial heterologous expression attempts in *E. coli* BL21 successfully yielded mycetohabin-15 (**19**) and mycetohabin-16 (**20**), while the burhizin-23 (**21**) construct (vector pEB45) produced only a truncated variant due to expression incompatibility. The structures of these three compounds as shown in Figure 2 and Figure 3. Considering host compatibility, the mushroom pathogen *Burkholderia gladioli* pv. *agaricicola* HKI0676 was selected as a heterologous expression host, which successfully generated the target compound. This study not only expanded the known structural diversity of lasso peptides but also established a critical precedent for host selection in heterologous expression systems, demonstrating that heterologous expression hosts can overcome expression barriers encountered in conventional bacterial platforms.

**Figure 4:**
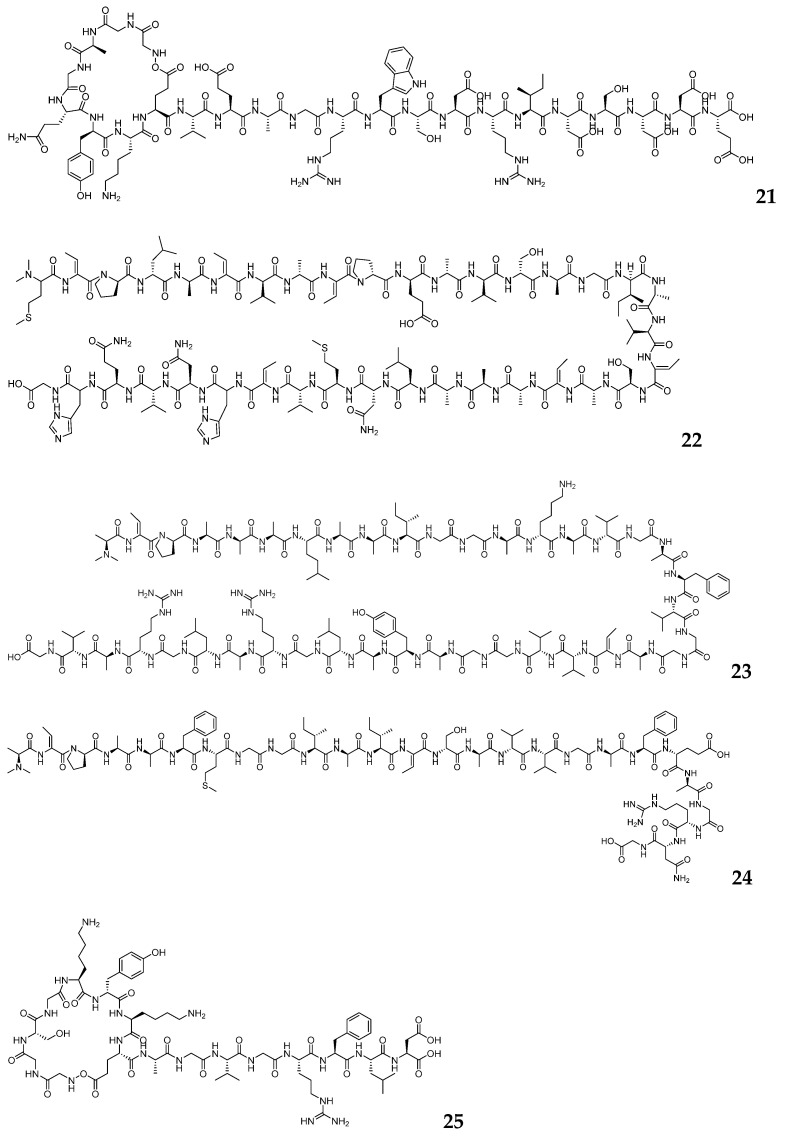
The chemical structures of RiPPs by genome mining (**21**–**25**).

Shang-Wen Luo and his team analyzed the genome of *Streptomyces yunnanensis* using antiSMASH, successfully identifying two linear bacteriocin BGCs, named *yan* and *ydn* [74]. During heterologous expression, *yan* and *ydn* were individually cloned into the *E coli-Streptomyces* shuttle vector pSET-kasO which contains a strong promoter, kasO, upstream of the cloning site to drive exogenous gene expression. Screening of different host strains and culture media ultimately enabled the expression of the *yan* in *Streptomyces azureus* M1154 and the *ydn* in *Streptomyces variegatus* GX28. This successfully resulted in the isolation of three linear anti-mycobacterial compounds, yunnanaridins A–C (**22**–**24**), which are shown in Figure 4. This approach demonstrated the critical importance of matching specific biosynthetic pathways with compatible expression hosts, as evidenced by the differential expression outcomes between the two gene clusters.

The Ion Torrent Personal Genome Machine (PGM) is a sequencing platform that operates on the principle of detecting changes in hydrogen ion (H^+^) concentration released during DNA synthesis, enabling high-throughput sequencing [75]. Rapid Annotation using Subsystem Technology (RAST) is an automated microbial genome annotation platform that utilizes the subsystem functional classification framework [76]. It facilitates gene function prediction through comparisons with known functional protein databases. Juan A. Asenjo and his team employed an integrated genomic approach to characterize the novel actinomycete *Streptomyces huasconensis* HST28T, combining Ion Torrent PGM with RAST [77]. This strategy enabled the identification of a compact lasso peptide biosynthetic gene cluster (*hpt*), whose heterologous expression produced huascopeptin (**25**), a novel Gly1-Asp7 macrocyclic lactam ring lasso peptide, which is shown in Figure 4. Remarkably, huascopeptin (**25**) represents the smallest known lasso peptide to date, featuring an unusually compact ring-tail architecture that defies conventional structural paradigms for this class of ribosomally synthesized peptides. This discovery not only expands the known size range and structural diversity of lasso peptides but also provides new insights into their structure–activity relationships.

### 2.2. Genome Mining of Terpenoids

Terpenoid genome mining employs distinct strategies for fungal and bacterial systems, focusing on key enzymatic signatures in their respective biosynthetic pathways [78]. The primary targets for terpenoid genome mining in fungi are typically individual TSs and prenyltransferase terpene synthases (PTTSs) [79]. The catalytic core of PTTSs comprises two or more independent terpene synthase modules, which mediate multi-step cyclization reactions to generate structurally complex hybrid terpenoids [80,81]. Bacterial terpenoid mining, conversely, emphasizes the identification of specialized post-modification enzymes that impart structural diversity to core terpene scaffolds.

**Figure 5:**
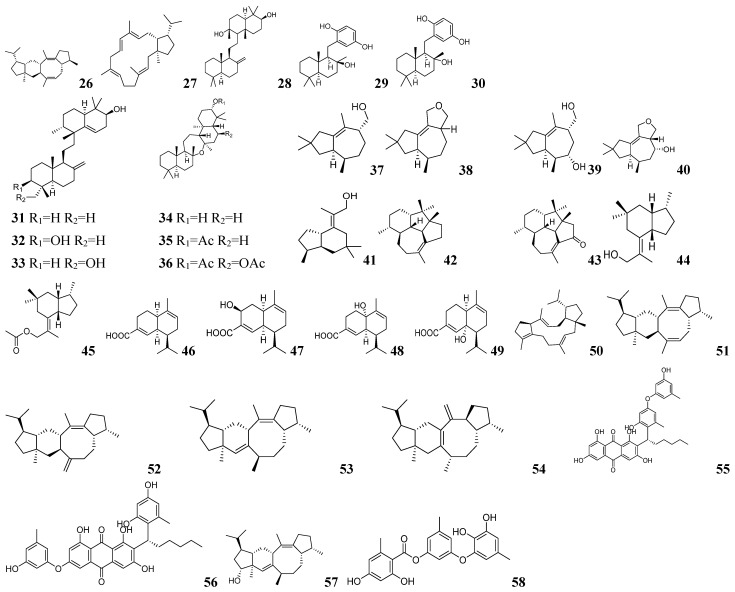
The chemical structures of terpenoids by genome mining (**26**–**58**).

HMMER is a widely used suite of tools for biological sequence analysis based on HMMs, enabling protein/nucleic acid sequence homology searches, gene family classification, and functional annotation [82,83]. Tian-gang Liu and his team developed an efficient workflow for discovering novel PTTS [80]. Firstly, using HMMER comprehensive searches across 519 fungal genomes in the Pfam database to identify sequences containing both PF03936 (prenyltransferase) and PF00348 (terpene synthase) domains—the characteristic signature of PTTS enzymes. Secondly, the team screened the NCBI and UniProt databases for PTTS genes, selecting hypothetical TSs with an amino acid sequence length of approximately 700 and conserved motifs within the PT and TS structural domains [84,85,86,87]. Combining these two datasets and retaining PTTS with less than 80% similarity, 74 candidate PTTS genes were identified. Functional characterization of the 74 candidate PTTS genes was performed using a high-efficiency precursor-providing yeast chassis coupled with a high-throughput automated platform. This system enabled rapid construction and analysis of numerous yeast mutants, revealing 34 functional PTTS enzymes including PTTS010 which produces sesterevisene (**26**), and PTTS009/037/054 which generates sesterorbiculene (**27**). The structures of these two compounds are shown in Figure 5. This study significantly accelerated the functional characterization of PTTSs by integrating a high-efficiency precursor-providing yeast chassis with a high-throughput automated platform, enabling the rapid construction of numerous yeast mutants from gene fragments. This versatile system is also applicable to the functional study of other terpene synthases.

Yudai Matsuda and his team developed FunBGCeX, an innovative fungal genome mining tool that addresses the challenge of identifying BGCs associated with domain-free enzymes [88]. The team first constructed a fungal BGCs (FunBGCs) database containing approximately 700 fungal BGCs, from which they extracted 5070 protein sequences for comprehensive Pfam domain analysis using HMMER. This analysis revealed 572 proteins lacking conventional domain architecture, highlighting a significant gap in current mining approaches. To overcome this limitation, the team developed specialized hidden Markov models (HMMs) targeting PKS and NRPS domains and constructed a DIAMOND database incorporating all biosynthesis-related proteins. Screening of BGCs encoding terpene cyclase Pyr4 homologs from 1990 fungal reference genomes was performed using FunBGCeX. Three BGCs (*homo*, *fumi*, and *alli*) encoding SHC/OSC-like proteins from different fungi and one BGC (*mos*) from *Neoarthrinium moseri* CBS 164.80 were identified through screening [89]. Nine new terpenoids (**28**–**36**) were produced by cloning the relevant genes into expression vectors and heterologously expressing them in *Aspergillus oryzae* NSAR1 or NSARU1. The structures of these compounds are shown in Figure 5. Unlike conventional mining software, FunBGCeX combines manually curated reference data with customized HMM profiling to specifically target atypical, domain-free enzyme-associated BGCs with superior precision and efficiency, establishing a new paradigm for uncovering fungal metabolic diversity.

There are many more studies of fungal TSs. After whole genome sequencing of *Irpex lacteus* by Guang-kai Bian and his team, gene cluster annotation was performed using antiSMASH [90]. Their integrated bioinformatics approach identified 19 potential sesquiterpene synthase (STS) genes, from which sequence comparison revealed 10 non-alleles. Heterologous expression of the selected *Il4946* gene in *Aspergillus oryzae* produced five new sesquiterpenoids: iltremulanols A–D (**37**–**40**) and trichobrasilenol (**41**), which are shown in Figure 5. Sheng-ying Li and his team identified a terpene BGC (*ven*) in *Streptomyces venezuelae* ATCC 15439 through gene annotation [91]. This BGC encodes the production of two diterpene compounds, namely venezuelaenes A–B (**42**–**43**), as shown in Figure 5, which feature a novel 5-5-6-7 tetracyclic backbone. Using known PaFS and AcOS sequences as probes, Jaclyn M. Winter and his team identified a cluster of potential bifunctional TS genes (*tnd*) in the genome of *Aspergillus flavipes* CNL338 [92]. This gene cluster encodes the production of a novel diterpene, namely talarodiene, which features a unique tricyclic structure. Tian-gang Liu and his team sequenced and annotated the genome of *Trichoderma viride* J1-030, identifying the potential TS gene *Tvi09626* through screening [93]. The gene catalyzed the production of a novel five or six bicyclic sesquiterpene (**44**) and its esterified derivatives (**45**), which are shown in Figure 5. This is the first sesquiterpene synthase characterized in this fungus, enriching the diversity of terpenoids in *Trichoderma viride*.

CD-HIT (Cluster Database at High Identity with Tolerance) is a widely used bioinformatics tool for clustering and comparing protein or nucleotide sequences [94,95]. BiG-SCAPE (biosynthetic gene similarity clustering and prospecting engine) is a bioinformatics tool designed for analyzing and comparing BGCs [96]. It performs cluster analysis based on sequence similarity and gene function to identify groups of genes with similar biosynthetic potential. Pei-Yuan Qian and his team utilized CD-HIT and BiG-SCAPE to comprehensively screen NCBI bacterial genomes and identified 2892 cytochrome P450-containing terpene synthase/cyclase genes that were organized into 355 distinct cluster families [97]. Focusing on the phylogenetically unique *lev* gene cluster that formed a discrete group in SSN, were selected for heterologous expression in *Streptomyces azureus* M1146. This led to the production of four novel α-amorphene type sesquiterpenes, namely levinoids A–D (**46**–**49**), as shown in Figure 5. Similarly, Kui Hong and his team identified the candidate gene *Au11189* from the *Aspergillus ustus* 094102 genome through BLAST search and functional prediction, naming it *AuAS* [98]. Initial heterologous expression of *AuAS* in *Aspergillus oryzae* NSAR1 produced five novel sesquiterpenes, namely aspergiltriene A (**50**) and aspergildienes A–D (**51**–**54**), while subsequent co-expression with the adjacent cytochrome P450 gene *AuAP450* yielded four structurally distinct dibenzoterpenoid alcohols aspergilols A–D (**55**–**58**). The structures of these compounds are shown in Figure 5. This method focuses on genome mining of modifying enzymes, overcoming the limitation of relying solely on TSs for novel compound discovery. It provides a more efficient approach to identify new bacterial terpenoids.

### 2.3. Genome Mining of Polyketides

**Figure 6:**
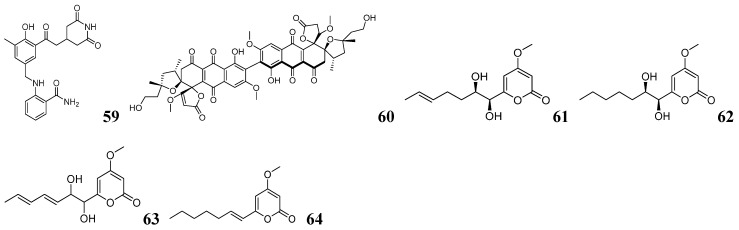
The chemical structures of polyketides by genome mining (**59**–**64**).

Seq2PKS is a machine learning-based algorithmic tool developed by Hosein Mohimani and his team for predicting the chemical structures of type I *cis*-acyltransferase (AT) polyketide compounds [99]. Its specific functions include the following: (1) predicting compounds produced by type I PKS gene clusters and improving prediction accuracy through mass spectrometry database calibration; (2) predicting acceptor substrate ranges of AT domains using an ExtraTrees-based classification algorithm, achieving an overall accuracy of 94%; (3) predicting the biosynthetic logic of *cis*-AT polyketides using a paired nearest neighbour (PNN)-based search combined with gene order in the genome [100]; (4) predicting polyketide core structure post-modification using a custom enzymatic modification database. When applied to analyze *Streptomyces actiphen* genomes from the NRRL collection, Seq2PKS identified a novel long-chain polyketide system with 55% similarity to known actiphenol biosynthetic pathways generating 258 structural predictions. Using the in-house Dereplicator+ tool, the team identified two mass spectrometry matches to polyketides, including the novel compound 2-aminobenzamide-actiphenol (**59**) upon expression, as shown in Figure 6 [101]. The algorithm integrates mass spectrometry data to predict structures of diverse mature polyketides, including those with unknown modifications. It outperforms PRISM and antiSMASH in benchmarking for *cis*-AT polyketide BGC prediction.

Hui-Ming Ge and his team screened 21,728 actinomycete genomes (NCBI database and in-house sequencing data) via BLAST 2.14.0, identifying 5944 type II PKS and 919 KAS III-containing gene clusters [102]. Two homologous gene clusters (*spi* and *msp*) from *Streptomyces spectabilis* NA07643 and *Micromonospora* sp. HM134 were finally identified. AntiSMASH annotation of the *spi* gene cluster revealed genes encoding type I PKS, type II PKS, KAS III, and multiple modifying enzymes, enabling the successful heterologous expression of spirocycline A (**60**) as shown in Figure 6. Spirocycline A (**60**) showed significant inhibition against *Micrococcus garciniae* (MIC = 2 μg/mL). Genome sequencing of the endophytic fungus *Calcarisporium arbuscula* by Ling Liu and his team resulted in the identification of 68 potential BGCs, including a highly reduced polyketide synthase (HRPKS) cluster *cpn* [103]. Heterologous expression of constructed plasmids containing *cpnA*, *cpnB*, and *cpnC* in *Aspergillus oryzae* A1145 yielded the novel α-pyranones calcapyrones A–B (**61**–**62**), along with biosynthetic intermediates calcapyrones C–D (**63**–**64**), as shown in Figure 6. Calcapyrone C (**63**) is weakly cytotoxic to cancer cells.

## 3. Metabolome Mining NPs

### 3.1. Metabolome Mining Based on MS

**Figure 7:**
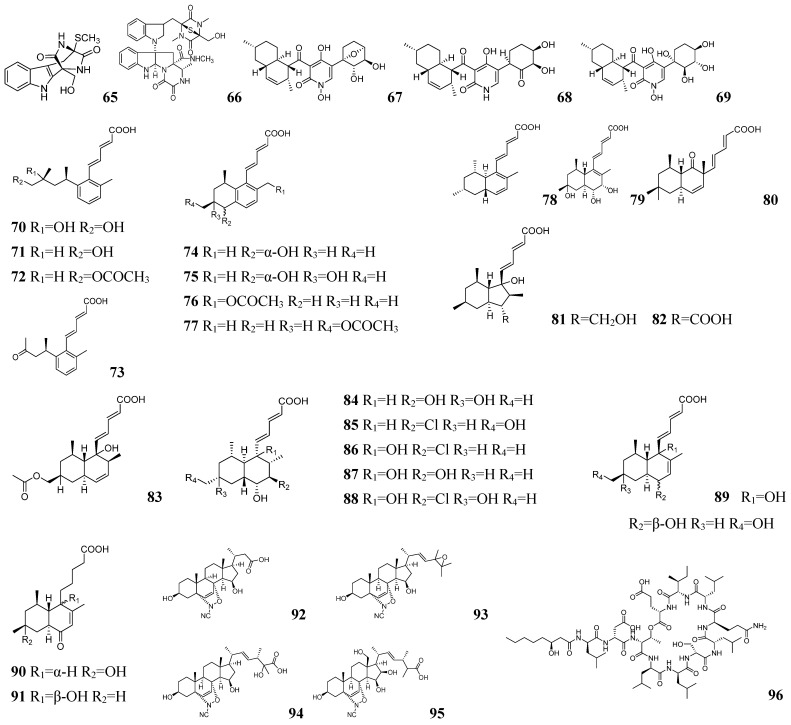
The chemical structures of NPs by metabolome mining based on MS (**65**–**96**).

Ultra-performance liquid chromatography coupled with quadrupole-time-of-flight tandem mass spectrometry (UPLC-Q-TOF-MS/MS) is a high-resolution analytical technique for accurate compound identification and quantification [104]. UPLC significantly reduces analysis time and improves sensitivity. The Q-TOF system fragments ions via CID and screens target ions by interpretation of *m*/*z* patterns to infer structural features [105]. UPLC-Q-TOF-MS/MS is the leading technique for metabolomics, unknown compound identification, and precise quantification, combining ultrafast separation with high-resolution mass detection. Gang Ding and his team used UPLC-Q-TOF-MS/MS to screen for compounds containing three characteristic fragments when mining secondary metabolites from *Chaetomium cochliodes* [106]. Previous studies confirmed that chetomin analogs with polysulfide bridges are cyclic peptides exhibiting an asymmetric dimeric structure. The key biosynthetic step in chetomin analog production is dimerization, which generates numerous characteristic fragment ions. The mass spectra of chetocochliodins C/D/E reveal shared characteristic fragments at *m*/*z* 270, *m*/*z* 284, and *m*/*z* 282. Structural analysis revealed these fragments derive from either (1) HOCH_3_/HSCH_3_ elimination from protonated molecules or (2) sequential loss of two HOCH_3_/HSCH_3_ units, enabling classification into three structural subtypes (A–C). Using UPLC-Q-TOF-MS/MS to target compounds containing the three characteristic fragments, the team successfully identified two novel chetomin analogs, namely chetocochliodins M (**65**) and N (**66**), as shown in Figure 7. Chetocochliodin N (**66**) demonstrates potent cytotoxicity against A549 and HeLa cancer cell lines, exhibiting superior activity compared to the positive control cisplatin. These findings highlight its potential as a lead compound for anticancer drug development. The results further highlight the crucial role of sulfur bridges in mediating the cytotoxicity of chetomin analogs, providing a foundation for investigating their structure–activity relationships.

GNPS is an open-access platform that utilizes MS data for molecular network analysis and natural product annotation. GNPS has three main advantages: (1) free and open source, which reduces the cost of scientific research; (2) a powerful public database with wide coverage of compound types; (3) richer data shared by scientists worldwide. The GNPS workflow is straightforward: users simply convert LC-MS/MS data to .mzXML format and upload the files to the platform (https://gnps.ucsd.edu, accessed on 15 March 2025), which automatically generates interactive molecular networks for visualization and analysis. GNPS is a powerful tool in metabolomics with a wide range of applications. Jun-Feng Wang and his team employed a targeted metabolomics approach to discover novel 4-hydroxy-2-pyridone alkaloids from sponge-derived *Arthrinium arundinis* ZSDS1-F3 [107]. By integrating LC-MS/MS analysis with GNPS molecular networking, they identified a characteristic cluster containing known arthpyrone alkaloids C and G, which guided the isolation of three structurally novel analogs—arthpyrones M–O (**67**–**69**) as shown in Figure 7. Arthpyrones M–O (**67**–**69**) demonstrated cytotoxic activity against some cancer cell lines (IC_50_ = 0.26–6.43 μM). Arthpyrone O (**69**) had strong inhibitory and pro-apoptotic effects on SCLC cell lines in vitro and significantly inhibited SCLC cell xenograft tumor growth in in vivo experiments. This study identifies promising new drug candidates for SCLC therapy while expanding the potential anticancer applications of 4-hydroxy-2-pyridone alkaloids.

**Figure 8:**
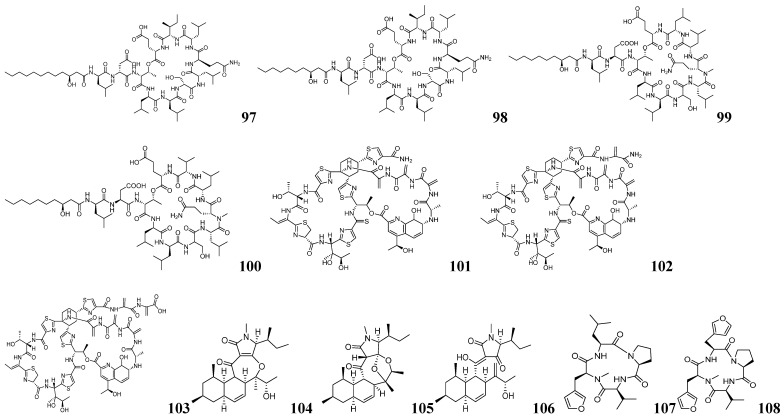
The chemical structures of NPs by metabolome mining based on MS (**97**–**108**).

Yong-Hong Liu and his team employed the GNPS platform to explore tanzawaic acid (TA) derivatives from *Penicillium steckii* SCSIO 41025 [108]. They successfully isolated and identified 40 TA derivatives, including 22 novel compounds (**70**–**91**), as shown in Figure 7. Most of these compounds inhibited LPS-induced NF-κB activity and RANKL-induced osteoclast differentiation. Christine Beemelmanns and his team employed LC-MS coupled with GNPS-based metabolomics to isolate four novel ergosterol derivatives, namely podaxisterols A–D (**92**–**95**), as shown in Figure 7 from *Podaxis* sp. Ethiopia [109]. During metabolomic analysis of *Pseudomonas* spp. strain FhG100052, Jens Glaeser and his team identified a dense cluster of cyclic lipopeptides (CLPs) exhibiting rich molecular networking features [110]. Through optimization of culture conditions, they successfully isolated five novel CLPs: stechlisin B2 (**96**), stechlisin F (**97**), tensin (**98**), stechlisin D3 (**99**), and stechlisin C3 (**100**). The structures of these compounds are shown in Figure 7 and Figure 8. The antimicrobial activity of stechlisins against *Moraxella catarrhalis* FH6810 exhibited a strong correlation with lipid chain length. Specifically, stechlisin B2 (**96**) had no inhibitory effect, tensin (**98**) had moderate activity, and stechlisin F (**97**) had significant activity. Christine Bemelman and his team discovered that *Amycolatopsis saalfeldensis* inhibited *Pseudoxylaria* sp.X802 in co-culture [111]. GNPS analysis of large-scale cultures yielded three novel type II thiopeptides, namely saalfelduracins B–D (**101**–**103**), as shown in Figure 8, which showed antimicrobial activity against Gram-positive bacteria.

FBMN is an advanced LC-MS/MS analysis method that enhances molecular network accuracy and quantification by integrating MS^1^ feature peaks with MS^2^ spectra. Compared with traditional GNPS, FBMN has four main advantages: (1) it avoids erroneous clustering due to retention time drift in traditional GNPS through RT correction and peak alignment and can distinguish isomers; (2) it correlates MS^1^ peak intensities, which allows cross-sample comparisons such as metabolite differences in different culture conditions; (3) unlike traditional GNPS that mainly relies on DDA (data dependent acquisition), FBMN can handle DIA (data non-dependent acquisition) to improve the detection rate of low abundance compounds [112,113]; (4) FBMN can integrate tools such as SIRIUS for more accurate structure prediction. In their investigation of secondary metabolites from *Epicoccum* sp. 1-042, Yun-Ying Xie and his team performed LC-MS/MS analysis of fractionated extracts [114]. The MS data were processed using MZmine, analyzed via FBMN on the GNPS platform, and visualized with Cytoscape software (v3.10.3). Polycyclic tetrahydropyrimidinic acids containing cis-decahydronaphthalene, namely epicolidines A–C (**104**–**106**), as shown in Figure 8 were isolated and identified, of which epicolidine B (**105**) and C (**106**) exhibited inhibitory activity against Gram-positive bacteria. In recent years, FBMN has evolved from primarily serving as a compound discovery tool to becoming increasingly valuable for metabolomic data processing and target compound prioritization.

Mass Spectrometry Query Language (MassQL) enables rapid extraction of target information from complex LC-MS/MS data [115]. The method enables automated, high-precision screening of target compounds through customizable parameters including parent ions, fragment ions, and neutral loss patterns. This automated analytical process effectively replaces traditional manual peak extraction and facilitates efficient batch processing of large datasets. Raphael Reher and his team developed an innovative mass spectrometry workflow combining MassQL with customized computational scripts to systematically identify compounds containing the diagnostic N-methyl-3-(3-furoyl)-alanine (NMefAla) fragment [116]. The approximate mining process is shown in Figure 3B. They employed MassQL’s targeted query capabilities to screen LC-MS/MS datasets for diagnostic fragment patterns, complemented by two specialized scripts—the first identifying NMefAla-containing structures and the second applying iminium ion filters to reduce false positives. Following GNPS-FBMN molecular networking, MassQL-enabled substructure annotation of network nodes facilitated the discovery of two novel proline-derived endocannabinoids, namely endolides E–F (**107**–**108**), as shown in Figure 8. Endolide F (**108**) has moderate antagonistic activity against arginine pressin V_1A_ receptors. While MassQL-annotated molecular networks enhance structural characterization by adding substructural information to facilitate novel compound identification, the approach presents several limitations: (1) demanding instrumentation requirements, and (2) inability to perform MS^2^ signal quantification.

### 3.2. Metabolome Mining Based on NMR

**Figure 9:**
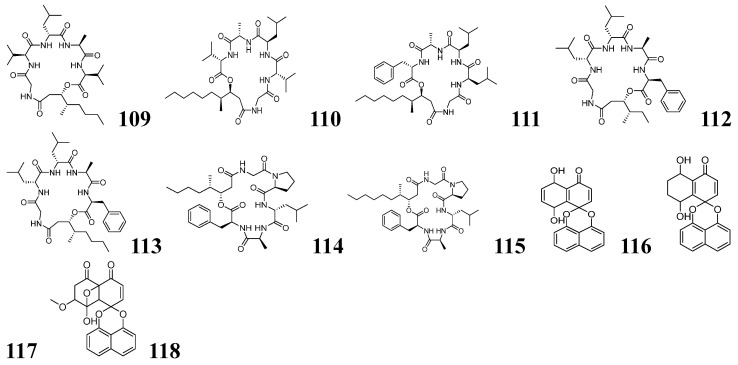
The chemical structures of NPs by metabolome mining based on NMR (**109**–**118**).

^1^H NMR is a powerful analytical technique with powerful capabilities for the structural identification of organic compounds, mixture composition analysis, and isomer differentiation. ^1^H NMR chemical shifts exhibit characteristic ranges that reflect distinct structural features: aliphatic protons (R-CH_3_): *δ_H_* 0.5–2.0 ppm; olefinic protons (=C-H): *δ_H_* 4.5–6.5 ppm; aromatic protons (Ar-H): *δ_H_* 6.5–8.5 ppm; carboxylic acid protons (-COOH): *δ_H_* 10–13 ppm. Hang-Lun Shao and his team pioneered an integrated analytical approach combining HPLC-MS/MS molecular networking with ^1^H NMR spectroscopy to discover novel bioactive peptides from 270 marine-derived Penicillium species [117]. Their workflow initially identified a distinctive metabolite cluster through characteristic neutral amino acid fragment losses in mass spectra, with subsequent ^1^H NMR analysis of target extracts revealing diagnostic amino acid signatures (exchangeable protons at *δ_H_* 9.00–7.50 and α-protons at *δ_H_* 4.50–3.50). Seven new peptide analogs chrysogeamides A–G (**109**–**115**) as shown in Figure 9 were isolated. The study establishes a robust template for accelerating the identification of bioactive NPs from complex microbial extracts by coupling molecular networking with NMR.

MADByTE is a mixture analysis method based on NMR and diffusion-ordered spectroscopy (DOSY), which is mainly used for the separation and component identification of complex mixtures [118,119,120]. The method calculates diffusion coefficients based on differential diffusion rates of molecules with varying sizes in solution, separates mixture components into distinct diffusion groups according to these coefficients, and subsequently identifies molecular structures within each group by integrating chemical shifts with coupling constant data. MADByTE enables component analysis of complex mixtures without physical separation. Nicholas H. Oberlies and his team used the MADByTE platform for dereplication in mining fungal metabolites for the resorcylic acid lactones (RALs) and spirobisnaphthalenes with good results [118]. By establishing a reference database containing heteronuclear single quantum coherence (HSQC) and total correlation spectroscopy (TOCSY) spectra of 19 RALs and 10 spirobisnaphthalenes, they enabled efficient on-target/off-target discrimination through MADByTE analysis. After investigating the structural features and bioactivities of RALs and spirobisnaphthalenes, an analysis of the full association network using the MADByTE platform showed that RALs with specific structures formed clusters in the bioactivity network, revealing a structure–activity relationship. The data from seven fungal extracts were analyzed in the above process, identifying strain MSX64790 as the target. Through combined mining using the MADByTE platform and 2D spectral analysis, three spirobisnaphthalene-like compounds palmarumycins CP20–CP22 (**116**–**118**) as shown in Figure 9 were obtained. This study not only marks the first successful implementation of MADByTE for fungal metabolite dereplication but also demonstrates its unique capability to visually map bioactivity patterns onto structural networks, establishing a powerful new paradigm for conducting SAR studies directly in complex mixtures.

## 4. Genomic and Metabolomic Guided the Isolation of NPs

### 4.1. Genomics Combined with Isotope Labeling and NMR

The landscape of bioinformatics tools for microbial natural product discovery features complementary platforms like antiSMASH and RODEO for BGC analysis, with antiSMASH 5.1 specifically enhancing RiPPs identification through improved cluster boundary prediction and recognition algorithms compared to its predecessors. Parallel developments include RIPPMiner, which adopts distinct computational approaches from RODEO while serving similar functions in RiPP-associated BGC prediction [121,122]. Isotope feeding experiments are a classical method for tracing metabolic pathways, elucidating biosynthetic mechanisms, and studying biochemical transformations using stable or radioactive isotope-labeled precursors [123]. Isotope feeding experiments combined with NMR and the above bioinformatics tools can efficiently resolve the synthesis logic and backbone structure of metabolites.

**Figure 10:**
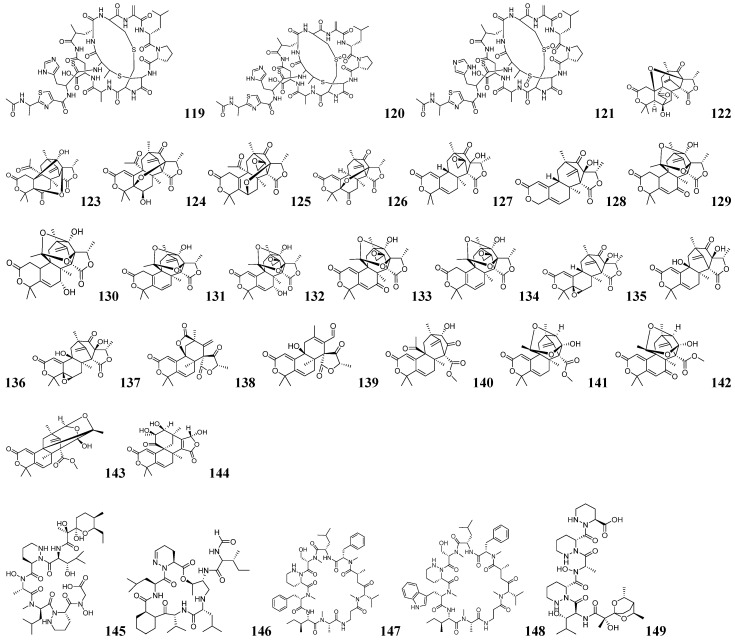
The chemical structures of NPs by integrated mining (**119**–**149**).

Harald Gross and his team developed an integrated approach combining bioinformatics prediction with advanced NMR-guided isotope tracing to discover novel RiPPs in *Nocardia terpenica* strains IFM 0406 and IFM 0706T [124]. Using antiSMASH 5.1, RODEO, and RIPPMiner, they identified the *nta* gene cluster encoding putative thiazole/oxazole-modified RiPPs. To characterize the metabolites encoded by the *nta* gene cluster, isotope feeding experiments were performed using (^15^NH_4_) _2_SO_4_, [^2^H_10_]-L-leucine, and [^2^H_7_]-L-proline. The results indicated that the target compound is a 12- to 13-amino acid polypeptide. Structural analysis, combined with genomic data on characteristic enzymes, revealed the presence of thiazole/oxazole moieties. Targeted screening was performed for compounds with molecular weights >1300 Da exhibiting characteristic ^1^H-^13^C HMBC correlations of oxazole (*δ_H_* 8.18/*δ_C_* 148.32) or thiazole (*δ_H_* 8.18/*δ_C_* 174.91) moieties. Three new RiPPs were successfully isolated from *N. terpenica* IFM 0406 and 0706 and named nocathioamides A–C (**119**–**121**) as shown in Figure 10. This study utilized bioinformatics tools to predict the structural features of nocathioamides. By integrating stable isotope labeling with ^1^H-^13^C-HMBC analysis successfully identified the target peptides from complex metabolite mixtures, thereby resolving the key challenge of linking biosynthetic genes to their metabolic products in RiPP research. This approach is applicable not only to thiazole-containing RiPPs in Nocardia, but can also be adapted to discover other RiPPs with rare functional groups. The integration of ^1^H-^15^N-HSQC screening allows the study of imine-containing RiPPs; the adjustment of ^1^H-^13^C-HMBC sequences can tap into more thioamidated RiPPs, which provides a general idea for the study of RiPPs.

The SMART system (Secondary Metabolite Analysis Shell for Rapid Target identification) is a bioinformatics tool for microbial secondary metabolite target mining focused on the rapid identification and analysis of potential natural product BGCs [125]. It combines genome mining and metabolic network analysis strategies to accelerate the discovery and engineering of NPs. Ai-Li Fan and her team identified the *dhi* in the Antarctic fungus *Penicillium purpurogenum* AN13, which encodes dhilirane-type meroterpenoid (DM) analogs featuring a 3,5-dimethylorsellinic acid (DMOA) backbone [126]. A small-scale fermentation of *P. purpurogenum* AN13 was performed. Crude extracts were analyzed by NMR and LC-MS/MS, with HSQC data uploaded to the SMART system. Integration of GNPS and SMART analysis led to the identification of seven new DMs, namely dhilirolides O–U (**122**–**128**), as shown in Figure 10. SSN analysis of the key oxidase DhiD, which clusters with characterized α-KG-dependent oxygenases despite lacking close homology to known DMOA-pathway enzymes, demonstrated its exceptional catalytic versatility in generating structural diversity—a capacity confirmed through functional characterization that yielded 16 additional novel DMs (**129**–**144**) as shown in Figure 10. This research demonstrates how the SMART system’s dual genome-metabolome analysis capability can accelerate NP discovery by efficiently connecting biosynthetic genes to their metabolic products.

Dong-Chan Oh and his team developed an effective strategy combining genomic and metabolic spectroscopic features to target the mining of piperazinic acid (Piz)-containing NPs [127]. For targeted genome mining, they designed degenerate primers (pizKtzI and pizKtzT) targeting conserved regions of the KtzT and KtzI biosynthetic genes. From 2020 screened strains, they identified 62 putative Piz-producing candidates. Phylogenetic analysis of KtzT/KtzI amplicons clustered these strains into distinct evolutionary clades, enabling branch-specific prediction of potential Piz-containing compound structures. Five representative strains—*Streptomyces* GB16, GSM11, PC5, SNJ018, and BYK1239—were selected as targets for further analysis. For metabolomic characterization, the screening strains were cultured with ^15^NH_4_Cl to isotopically label potential Piz compounds. Subsequent analysis by ^1^H-^15^N-HSQC, HSQC-TOCSY, and HMBC confirmed the presence of Piz metabolites. Using the above strategy to isolate and characterize polyoxyperuin B seco acid (**145**), depsidomycin D (**146**), and lenziamides A–B (**147**–**148**) as shown in Figure 10, Lenziamide A (**147**) has significant antiproliferative activity against colon cancer cells and overcomes 5-FU resistance. This study establishes a genomic and spectral characterization-based research approach that enables the targeted discovery of novel NPs containing specific structural motifs from microorganisms lacking complete genome sequences, overcoming the limitations of traditional methods.

**Figure 11:**
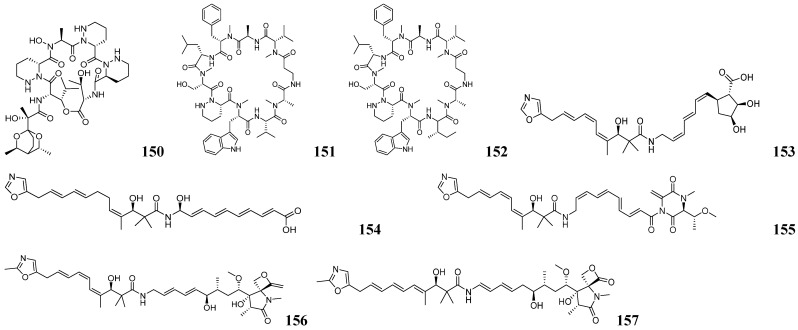
The chemical structures of NPs by integrated mining (**150**–**157**).

Genome mining combined with NMR-guided strategies has wide applications in targeting the isolation of Piz-like compounds. Raymond J. Andersen and his team screened the NCBI database using the BLAST-P program with the amino acid sequence of the Piz synthase KtzT as a probe, ultimately selecting *Streptomyces incarnatus* NRRL 8089, a strain exhibiting over 50% homology [128]. The metabolic processes were labeled and analyzed by ^15^N NMR, including ^1^H-^15^N-HSQC and ^1^H-^15^N-HSQC-TOCSY spectra of the crude extracts. The characteristic signals (*δ_N_* 299.8 ppm and *δ_H_* 4.52 ppm for N-H) and their associated spin systems in the Piz compounds guided a hierarchical separation, yielding the target incarnatapeptins A (**149**) and B (**150**) as shown in Figure 10 and Figure 11. Similarly, Hua-Yue Li and his team analyzed the ^1^H NMR data of the marine actinomycete *Streptomyces* sp. S063 and identified a characteristic negative hydrogen signal attributable to Piz [129]. Further analysis with antiSMASH 6.0 and SSN identified the Piz-related BGCs in this strain. Through NMR-guided stepwise isolation, two Piz-containing cyclic decapeptides, namely lenziamides D1 (**151**) and B1 (**152**), were obtained as shown in Figure 11. Combining genome mining with screening methods—including characteristic NMR signals and ^15^N NMR—enables targeted isolation of low-abundance Piz-containing NPs, thereby expanding their structural diversity.

**Figure 12:**
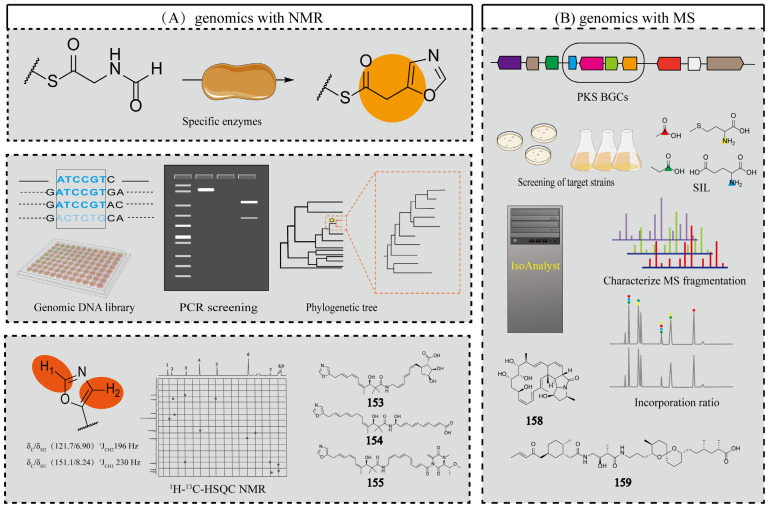
(**A**) Genomics and NMR aid in mining new NPs. (**B**) Genomics and MS aid in mining new NPs.

Dong-Chan Oh and his team have developed a new strategy that combines genomic characterization with spectral characterization to efficiently discover NPs containing specific structural motifs without the need for isotope labeling [130]. The specific mining process is shown in Figure 12A. During genomic characterization, PCR primers containing concatenated motifs (SGGKDS) were designed to screen for oxazole cyclase homologs, using known terminal oxazole compounds (inthomycin B and phthoxazolin) as references. In the genomic screening stage, degenerate PCR primers containing motifs (SGGKDS) were designed for screening the oxazole cyclase homologous genes based on the known terminal oxazole compounds inthomycin B and phthoxazolin. An in-house bacterial genomic DNA library of 1000 strains was screened and 16 hit strains were obtained and phylogenetically analyzed. To monitor oxazole production during small-scale fermentation, ^1^H coupling was disabled during ^1^H-^13^C HSQC acquisition, recording only ^1^*J_CH_* values and single-bond ^1^H-^13^C correlations. The presence of oxazoles was confirmed by detecting characteristic single-bond ^1^H-^13^C correlations at *δ_C/H_* 151.1/8.24 (^1^*J_CH_* = 230 Hz) and *δ_C/H_* 121.7/6.90 (^1^*J_CH_* = 196 Hz) in the ^1^H-^13^C HSQC spectra. Five terminal oxazole compounds lenzioxazole (**153**), permafroxazole (**154**), tenebriazine (**155**), and methyl-oxazolomycins A–B (**156**–**157**) were isolated using ^1^H-^13^C HSQC-guided fractionation as shown in Figure 11. This integrated approach combining genomic screening with ^1^H-^13^C HSQC analysis enables efficient detection of terminal oxazole compounds without isotopic labeling. The method can be extended to identify other heterocyclic compounds, including discrimination between imidazoles and oxazoles, demonstrating broad applicability.

### 4.2. Genomics Combined with MS

MS is a powerful metabolomics tool offering high sensitivity, resolution, and rapid analysis. In 2014, Douglas A. Mitchell and his team successfully identified cyclo-thiazomycin C through an approach combining genomic screening with MS detection [131]. The core innovation of this research lies in employing genome mining to identify microbial strains capable of producing dehydroamino acids (DHAAs). Treatment of extracts with dithiothreitol (DTT) resulted in a 154.0 Da mass increase of DTT adducts compared to the output metabolite, a feature that can be targeted to track DHAAs analogs using MS monitoring. This study demonstrates that the integrated use of genomics and MS constitutes an efficient strategy for novel NPs discovery.

**Figure 13:**
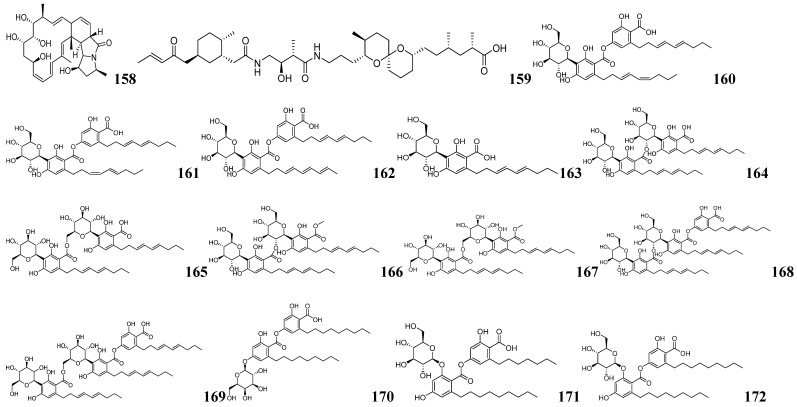
The chemical structures of NPs by integrated mining (**158**–**172**).

IsoAnalyst is an HR-MS-based software for isotopic fine structure (IFS) analysis. It can analyze isotope peak intensity ratios to determine the most probable molecular formula, while cross-referencing multiple databases enhances identification accuracy [132]. Roger G. Linington and his team utilized the IsoAnalyst platform to create metabolite-BGC mappings associated with BGC-based chemical structure predictions. The specific mining process is shown in Figure 12B. Specifically, four universal precursors—[1-^13^C] propionate, [1-^13^C] acetate, [1-^15^N] glutamate, and [methyl-^13^C] methionine—were used as stable isotope labeling (SIL) carriers to participate in biosynthetic processes as substrates. A control group without any additions was established to compare the MS data before and after treatment. The data were processed using IsoAnalyst to compare mass isotope distributions between labeled and unlabeled conditions, determining the degree of labeling for each precursor. Simultaneously, the strain genome was mined for BGCs using antiSMASH. Substrates for each BGC were predicted by integrating the MIBiG database with literature-derived information, generating a substrate prediction table. SIL precursor incorporation patterns were manually compared to theoretical incorporation rates predicted from BGC annotations to identify candidate BGCs associated with each labeled metabolite. The complete genome of *Micromonospora* sp. was analyzed using antiSMASH, and BGCs were manually curated for substrate prediction, generating a table of predicted BGC markers. The 246 MS features labeled under two or more SIL conditions were processed in IsoAnalyst, filtered to 100 based on peak type and intensity, and categorized into two broad groups according to their isotopic labeling patterns. Comparison with the annotated BGC list revealed that one of the most promising classes was associated with BGC *30c*. BGC *30c* was identified as responsible for the biosynthesis of lobosamide-like compounds. The predominant labeled metabolites in this group exhibited characteristic *m*/*z* profiles matching the lobosamide family, including distinctive [M+H−H_2_O]^+^ fragment ions generated through intragenic cleavage. Based on these findings, subsequent isolation and purification yielded two known lobosamide analogs and a new natural product, which was designated lobosamide D (**158**) as shown in Figure 13.

Roger G. Linington and his team also used IsoAnalyst to discover the new polyketide compound lagriamide B (**159**) from a Burkholderiales strain as shown in Figure 13 [133]. The researchers first sequenced 115 *Burkholderia cepacia* strains and conducted bioinformatics analysis using antiSMASH 6.0, identifying a gene cluster homologous to the known antifungal polyketide lagriamide A BGC. They screened the NCBI database for additional strains containing this conserved gene cluster. Building on the structural features of lagriamide A, parallel stable isotope labeling experiments were conducted using [1-^15^N] glutamic acid as the SIL substrate in two strains harboring the target BGCs. IsoAnalyst was employed to preferentially screen for molecules containing the target elements or structural motifs. Lagriamide B (**159**) was successfully identified. The IsoAnalyst platform enables the classification of specific metabolites through SIL and facilitates the correlation of these compounds with candidate BGCs. IsoAnalyst prioritizes compound mining based on biosynthetic relevance, employing a screening process that emphasizes biosynthetic relationships. Notably, it can identify biogenetic associations between molecules even when their spectral features differ significantly. This study demonstrates significant potential for application in the discovery of novel structural variants within compound families.

**Figure 14:**
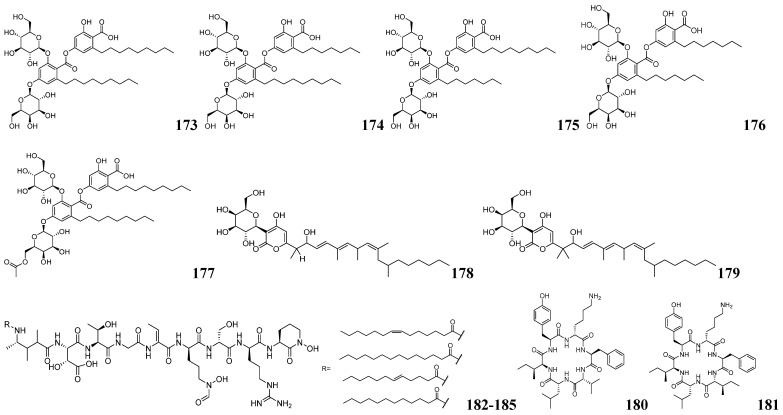
The chemical structures of NPs by integrated mining (**173**–**185**).

Jun-Gui Dai and his team developed an integrated approach combining enzyme-guided genome mining with MS-based metabolomics to discover multiple novel glycosylated NPs from fungal sources [134]. Functional characterization of stromemycin biosynthetic genes in *Aspergillus ustus* through in vivo gene knockout and heterologous expression revealed StmC as the responsible glycosyltransferase. Using AuCGT as a probe, they mined 158 homologous proteins and identified 80 candidate strains from NCBI and other databases. AntiSMASH analysis predicted that most AuCGT homologs co-localize with PKS clusters, suggesting their potential role in polyketide glycoside biosynthesis. To validate the polyketide glycoside synthesis potential of the candidate strains, seven fungal strains were preferred for fermentation culture, of which three strains were confirmed to possess the characteristic fragment ions of C-chain hexoses ([M−H−120]^−^ and [M−H−90]^−^). Through large-scale cultivation of *T. cellulolyticus*, 10 new glycosides, namely talarocellmycins A–C (**160**–**162**), carnemycin I (**163**), talarocellmycins D–G (**164**–**167**), and talarocellmycins H–I (**168**–**169**) were isolated. Eight novel glycosides, namely phaeomoniecin A (**170**), phaeomoniecin B–C (**171**–**172**), phaeo-moniecins D–G (**173**–**176**) and phaeomoniecin H (**177**), were obtained from *P. chlamydospora*. Two new α-pyran-3-C-β-D-galactoside compounds, verapyrones A–B (**178**–**179**), were obtained from *V. dahliae*. The structures of these compounds are shown in Figure 13 and Figure 14. This study also identified novel condensed phenolic acid C-/O-glycosyltransferases and α-pyranone C-glycosyltransferases, confirming that other fungal candidates, beyond the three mentioned strains, can also synthesize such glycosidic compounds. Genome mining with these novel glycosyltransferases could reveal more structurally distinct fungal glycosides.

Integrated genome mining and MS/MS analysis are widely used approaches for discovering novel NPs. Seung Hyun Kim and his team isolated nine bacterial strains from the sea pony epiphytic environment [135]. Among them, YSL2 exhibited the strongest inhibitory activity and high phylogenetic novelty, as a new species of the genus Nocardia, named *Nocardiopsis maritima* sp. nov. Strain YSL2 harbors 17 BGCs, one of which is linked to hexapeptide synthesis and may produce novel NPs. The hexapeptide analog maritiamides A–B (**180**–**181**) were successfully traced and characterized by LC-MS/MS-based molecular network analysis as shown in Figure 14. Similarly, Paul D. Boudreau and his team identified delftibactins BGC-homologous gene clusters in *D. lacustris* through whole-genome sequencing and antiSMASH analysis [136]. Iron restriction induced *D. lacustris* to produce novel lipophilic metallophores. Through MS-guided isolation, they obtained four structurally distinct delftibactin derivatives, namely delftibactins C–F (**182**–**185**), as shown in Figure 14 featuring modified lipid tails. This study employs an integrated metabolomics-genomics approach to systematically investigate bacterial specialized metabolites, elucidating both their chemical structures and biosynthetic pathways.

## 5. Discussion

This review comprehensively summarizes 185 new natural products discovered with genomics, metabolomics, and their integration strategies from 2018 to 2024, while highlighting key technological breakthroughs and emerging research frontiers. In RiPPs mining, tools such as MSSN and SPECO were used to link P450 enzymes to RiPPs. Subsequently, AlphaFold-Multimer was used to predict the enzyme–substrate binding conformation and validate the catalytic mechanism of P450 enzymes for RiPPs. The availability of multiple search tools for specific BGCs has allowed researchers to conduct more detailed targeted analyses of a particular class of BGCs of interest. These efforts are undoubtedly more detailed and in-depth compared to pre-2018 tools. For terpene discovery, HMMER screened fungal genomes for PTTS, while FunBGCeX optimized gene cluster boundary prediction, addressing pre-2018 limitations in non-model organism gene annotation. In polyketide mining, Seq2PKS predicted polyketide structures by integrating gene sequence–structure correlations, a strategy that expanded detectable BGC types compared to previous single-omics methods. This multi-dimensional validation (genomic prediction + metabolic profiling) minimized false negatives, enabling targeted isolation of low-abundance metabolites.

Metabolomics advancements, such as UPLC-Q-TOF-MS/MS, enabled high-resolution mass fragmentation for precise metabolite identification. The GNPS platform constructed molecular networks to cluster structurally related compounds, while FBMN (Fragmentation-Based Molecular Networking) incorporated retention time and peak intensity to resolve isomers—overcoming pre-2018 challenges in spectral ambiguity. SMAST (Structure Elucidation via Molecular Networking) directly linked NMR/HSQC data to BGCs, capturing mixture-specific features more efficiently than traditional metabolite tracing.

The integration of genomics and metabolomics strategies significantly improved NPs discovery efficiency through multidimensional technology synergies. We have summarized the genomics–metabolomics integration strategies mentioned in the text in a tabular format (as shown in Table 1) to show more clearly the workflow and their significant advantages. For example, in the discovery of nocathioamides A-C, RiPPs gene clusters containing thiazole/oxazole motifs were predicted for the first time from *Nocardia terpenica* genomes using antiSMASH and RODEO. Subsequently, stable isotope labeling was used to trace the peptide biosynthesis pathway, followed by identification and precise localization of the identified compounds by characteristic HMBC signals in complex metabolite mixtures. This integrated strategy not only overcomes the limitations of single-omics approaches for gene-metabolite associations, but also enables systematic metabolic pathway analysis through a closed-loop workflow combining bioinformatics predictions, spectroscopic validation, and isotope tracing. This approach accelerates the targeted discovery of low-abundance bioactive molecules and facilitates mechanistic elucidation. At the same time, several recent studies have shown that the integration strategy has facilitated the discovery of novel skeletons that have never been reported before, such as the discovery of the novel antifungal molecule mandimycin [54], the rare macrolide skeleton somalactams [137], and the non-squalene triterpene colleterpenol [138], to name just a few. The new discovery paradigm allows researchers to target the “dark matter” in microbial genomes, which will undoubtedly greatly accelerate the discovery of new skeletons and thus enrich the chemical diversity of natural molecules.

Although significant progress has been made for large-scale analysis of BGCs, direct prediction of chemical structures based on gene sequences is a difficult problem that is currently unattainable. The solution to this problem depends on further advances in synthetic biology, especially the accumulation of data for experimental validation of biosynthetic pathways. This will lead to an in-depth development of genomics analysis from BGCs type determination to compound structure prediction. MS-based metabolomics analyses also face the problem of false-positive detection signals and distorted prediction results. In addition to hardware performance upgrades, the performance of mass spectrometry fragment-based structural analysis may also be enhanced by AI-enabled big data modeling [139,140,141,142].

In summary, there is currently a profound paradigm shift in microbial natural products research, characterized by the emergence of synergistic, technology-driven discovery. While challenges in database integrity, strain culturability, and multi-omics integration remain, the combined power of these technological approaches has opened up unprecedented avenues for microbial chemical diversity. By refining predictive models, optimizing experimental workflows, and supporting interdisciplinary collaborations, the field is poised to overcome current limitations and deliver a new generation of bioactive molecules, cementing the central role of microbial natural products in drug discovery for decades to come.

## Figures and Tables

**Table 1:** The advantages of the integration of genomic and metabolomic strategies compared to before 2018.

Method	Workflow	Applications	Advancements
Genome mining combined with NMR-based isotope labeling	antiSMASH, RODEO and RIPPMiner combined prediction of RiPPs-type BGCs. Characteristic signals labeled by isotopes in ^1^H-^13^C HMBC spectra.	RiPPs with characteristic functional groups such as thiazole, oxazole, imine, and thioamide [124]. RiPPs with significant features in two-dimensional spectra are also applicable.	The comprehensive use of antiSMASH and various other tools enables more accurate identification of RiPPs BGC types.
Genome mining combined with SMART and molecular networking	Establishing links between compounds and BGCs using SMART analysis of NMR and HSQC data. Utilizing SSN to identify potential enzymes for subsequent expression.	DM class containing DMOA skeleton [125].	SMART has a stronger ability to capture characteristic information in mixtures and can directly associate compounds with BGC.
Genome mining combined with two-dimensional spectral features	Design primers to screen homologous genes. During the collection of ^1^H-^13^C HSQC, only the ^1^*J_CH_* value and the single bond correlation between ^1^H and ^13^C are recorded.	Terminal oxazole NPs [130]. NPs with special C-H correlations on ^1^H-^13^C HSQC.	Capturing single-key correlations enhances detection sensitivity, and a small trial is sufficient to determine whether a gene is successfully expressed.
Genome mining combined with isotope feeding and IsoAnalyst	Utilize antiSMASH 6.0 predictions and compare them with MIBiG to identify potential BGCs. Isotope-labeled precursor feeding. IsoAnalyst analyzes its biosynthetic pathway and the approximate skeleton of the compound.	Lobosamide-type polyene lactam [132]. Lagriamide polyketides [133].	IsoAnalyst can accurately capture the connections between molecules that are biologically related but have significantly different spectral characteristics.

## Data Availability

Not applicable.
